# The Effect of Various Types of Motorcycle Helmets on Cervical Spine Injury in Head Injury Patients: A Multicenter Study in Taiwan

**DOI:** 10.1155/2015/487985

**Published:** 2015-02-01

**Authors:** Carlos Lam, Mau-Roung Lin, Shu-Fen Chu, Shin-Han Tsai, Chyi-Huey Bai, Wen-Ta Chiu

**Affiliations:** ^1^Department of Critical and Emergency Medicine, Wan Fang Hospital, Taipei Medical University, Taipei 116, Taiwan; ^2^Graduate Institute of Injury Prevention and Control, College of Public Health and Nutrition, Taipei Medical University, No. 250, Wu-Hsing Street, Taipei 110, Taiwan; ^3^School of Public Health, College of Public Health and Nutrition, Taipei Medical University, Taipei 110, Taiwan; ^4^Department of Neurosurgery, Shuang Ho Hospital, Taipei Medical University, Taipei 235, Taiwan; ^5^Department of Emergency Medicine, College of Medicine and Shuang Ho Hospital, Taipei Medical University, Taipei 110, Taiwan; ^6^Department of Public Health, College of Medicine, Taipei Medical University, Taipei 110, Taiwan; ^7^Department of Neurosurgery, Wan Fang Hospital, Taipei Medical University, Taipei 116, Taiwan

## Abstract

*Introduction*. The relationship between cervical spine injury (CSI) and helmet in head injury (HI) patients following motorcycle crashes is crucial. Controversy still exists; therefore we evaluated the effect of various types of helmets on CSI in HI patients following motorcycle crashes and researched the mechanism of this effect. *Patients and Methods*. A total of 5225 patients of motorcycle crashes between 2000 and 2009 were extracted from the Head Injury Registry in Taiwan. These patients were divided into case and control groups according to the presence of concomitant CSI. Helmet use and types were separately compared between the two groups and the odds ratio of CSI was obtained by using multiple logistic regression analysis. *Results*. We observed that 173 (3.3%) of the HI patients were associated with CSI. The HI patients using a helmet (odds ratio (OR) = 0.31, 95% confidence interval (CI) = 0.19−0.49), full-coverage helmet (0.19, 0.10−0.36), and partial-coverage helmet (0.35, 0.21−0.56) exhibited a significantly decreased rate of CSI compared with those without a helmet. *Conclusion*. Wearing full-coverage and partial-coverage helmets significantly reduced the risk of CSI among HI patients following motorcycle crashes. This effect may be due to the smooth surface and hard padding materials of helmet.

## 1. Introduction

**Figure 1:**
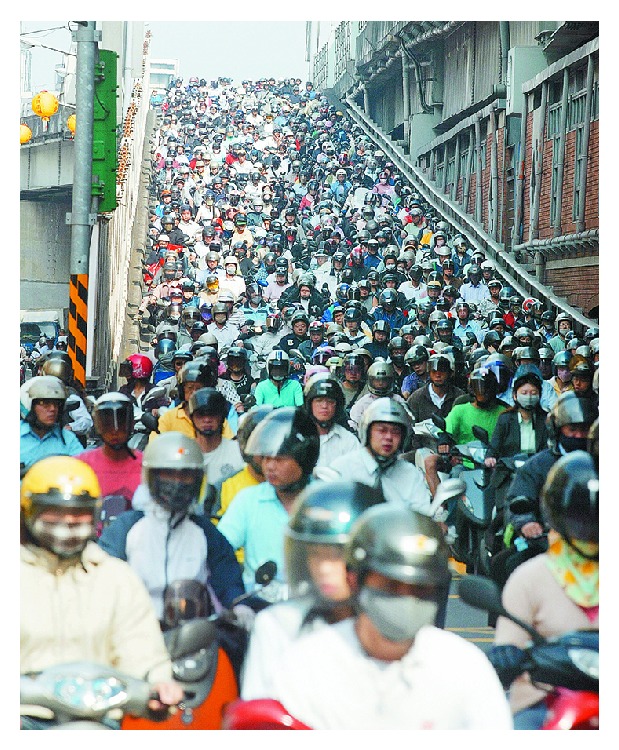
Rush hour motorcycle traffic in Taipei (permission to reproduce by http://udndata.com/).

Motorcycles are a crucial means of transportation in Taiwan (Figure 1). According to the Ministry of Transportation and Communications [1], 15 139 628 motorcycles were registered in 2012. Currently, there are 1.5 persons per motorcycle in Taiwan, overwhelmingly exceeding the motorcycle density in Vietnam and Malaysia (3 persons per motorcycle for both countries), Thailand (4 persons per motorcycle), Japan (10 persons per motorcycle), South Korea (26.7 persons per motorcycle), the United Kingdom (47.4 persons per motorcycle), and the United States (118.7 persons per motorcycle) [2].

Head injury has been regarded as a critical cause of death and disability among victims of motorcycle crashes [3–5], and previous reports have emphasized the close relationship between cervical spine injury (CSI) and head injury (HI) [6]. Despite the widespread agreement that helmets prevent head injuries, controversy still exists on the effect of helmet on CSI [7–10]. Crompton et al. (2011) utilized a large-scale study and concluded that motorcycle riders wearing helmets are less likely to suffer from CSI [11]. However, Crompton's study was not specific for HI patients and also admitted that the lack of helmet-type data was one of the limitations.

In Taiwan and other emerging economies people frequently used motorcycles as daily transportation tools, and the financial and social burden caused by HI and CSI following motorcycle crashes is important for both clinical physicians and public health providers. Therefore the issue of the effect of helmet on CSI was crucial for the victims of HI following motorcycle crashes. Because controversy still exists on the relationship between motorcycle helmet use and CSI, and which helmet type is superior, we utilized the data from Head Injury Registry in Taiwan and performed a multicenter case-control study to evaluate the effect of various types of motorcycle helmets on CSI in HI patients following motorcycle crashes and researched the mechanism of the effect of motorcycle helmets on CSI. The Institutional Review Board of Taipei Medical University granted approval for this study (Ref. number 201308033).

## 2. Patients and Methods

### 2.1. The Head Injury Registry in Taiwan

Data were extracted from the Head Injury Registry in Taiwan. This Head Injury Registry is an electronic database funded by the Taiwan Government since the 1990s that contains over 100 000 cases of HI in Taiwan [12]. Data were recorded by experienced physicians from 56 hospitals nationwide, covering 80% of major hospitals around the island. Because the Head Injury Registry concurrently provides demographic data, environmental factors, and clinical characteristics including severity, injury type, helmet type, treatment, and outcome, this database has been used in numerous published articles to explore various aspects of epidemiological and clinical topics related to HI and spinal injury [10, 13–21].

### 2.2. The Case-Control Study

Data provided by 25 hospitals in northern Taiwan were obtained from the Head Injury Registry and included in our study for analysis. To minimize the influence of the Taiwan Mandatory Helmet Law which was implemented nationwide in 1997, we only included the patients from 2000 to 2009. The inclusion criteria of this study were (1) patients with ICD-9 codes 800–804 and 850–854, covering the diagnoses of brain concussion, intracranial hemorrhage, and skull-bone fracture; (2) motorcycle crash; and (3) being over 17 years of age. Any cases with missing data on helmet use, helmet type, or CSI were excluded.

The occurrence of CSI after motorcycle crashes was the outcome variable in our study. CSI was defined as cervical spinal fracture. The predictor variables in our study were helmet use and helmet types, including full coverage and partial coverage. A full-coverage helmet (also known as an open-face helmet) protects the ears, cheeks, and back of the head, but it lacks a lower chin bar. A partial-coverage helmet (also known as half-helmet) features the same front design as a full-coverage helmet, but it lacks a lowered rear. The enrolled patients were divided into case group, consisting of patients with CSI, and control group, consisting of patients without CSI, and differences of motorcycle helmet use between case and control groups, including various helmet types, were compared.

Fourteen crucial demographic and clinical variables described by previous studies were included as covariates for testing the construct validity of the study [10, 12, 22–24]. The demographic variables included sex and age. The clinical variables included position on the motorcycle (driver or passenger), method of transportation to the hospital (self-transportation or ambulance delivery), hospital transfer (whether patients were transferred from another hospital), the Glasgow Coma Scale, HI-induced neurological impairment, thoracic spine injury, lumbar spine injury, skull-bone fracture, facial-bone fracture, chest injury, abdominal injury, and history of hospitalization caused by HI in the past 5 years.

### 2.3. Statistical Analysis

We first performed univariate analysis to assess the differences in helmet use, helmet types and above-mentioned covariates between case and control groups. Then we conducted multiple logistic regression analyses to assess the association of CSI and motorcycle helmet use as well as helmet types in HI patients after adjusting the effect of the above-mentioned covariates. Given the collinearity between helmet use and helmet types (*r* = 0.75; *P* < 0.001), these 2 variables were analyzed using separate models (Model 1 and Model 2) while the remaining variables were held constant in both models. These 2 models were used to estimate odds ratios (OR). SPSS Version 18.0 (SPSS Inc., Chicago, IL) statistical software was used to perform all statistical analyses. A *P* value less than 0.05 was considered statistically significant.

## 3. Results

**Figure 2:**
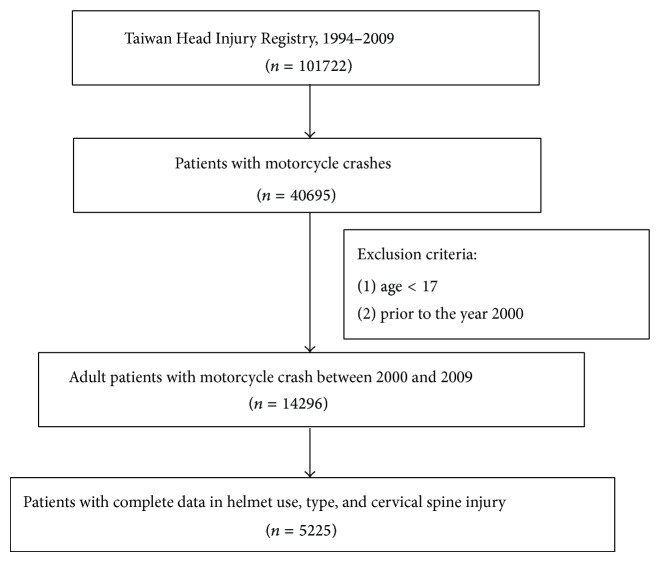
Patients' selection in the Head Injury Registry in Taiwan.

A total of 5225 eligible patients were divided into case and control groups for comparison according to whether CSI was present. One hundred seventy-three cases (3.3%) with CSI were included in the case group, whereas the remaining 5052 cases (96.7%) without CSI constituted the control group (Figure 2).

Table 1 showed the distribution based on helmet use, helmet type, and other crucial demographic and clinical variables between the case and control groups. Compared with the control group, those with CSI (case group) were more likely to be men (70.5% versus 61.8%) and those who failed to use helmets (23.7% versus 8.1%). When comparing the clinical variables, the case group exhibited injuries that were more severe than those of the control group, including hospital arrival by ambulance (91.4% versus 81.9%), HI-induced neurological impairment (15.9% versus 7.8%), thoracic spine injury (5.8% versus 1.0%), lumbar spine injury (2.9% versus 0.4%), chest injury (13.9% versus 7.9%), and abdominal injury (3.5% versus 2.4%).

Table 2 showed the result of multiple logistic regression analysis (Model 1). The HI patients who used a helmet exhibited a significantly decreased rate of CSI than that of patients who did not use a helmet (OR = 0.31, 95% CI = 0.19–0.49). Other significant risk factors included hospital arrival by ambulance (OR = 1.99, 95% CI = 1.08–3.67), thoracic spine injury (OR = 4.70, 95% CI = 2.00–11.05), and lumbar spine injury (OR = 4.82, 95% CI = 1.47–15.86).

In Table 3, Model 2 showed that HI patients who used a full-coverage helmet (OR = 0.19, 95% CI = 0.10–0.36) or a partial-coverage helmet (OR = 0.35, 95% CI = 0.21–0.56) experienced a significantly lower rate of CSI than that of patients who did not use a helmet. Other significant risk factors included thoracic spine injury (OR = 4.46, 95% CI = 1.89–10.48) and lumbar spine injury (OR = 4.87, 95% CI = 1.46–16.23). In order to directly compare the effect on CSI between full-coverage helmet and partial-coverage helmet, we repeated the comparison by choosing the patients with partial-coverage helmet as reference, and the results were shown as follows: OR = 0.55 (95% CI = 0.33–0.93) and 2.88 (1.78–4.67) for full-coverage helmet and nonhelmet patients, respectively. These results were consistent with the original results. We also reported no issue of collinearity in both Models 1 and 2 because the variance inflation factors among all covariates were less than 2.

## 4. Discussion

Previous studies reported that CSI occurs in 4%–8% of patients with HI [25–27]. In this study, the incidence of CSI in HI patients caused by motorcycle crashes was 3.3%. Our study confirmed that motorcycle helmets reduced the risk of CSI in HI patients following motorcycle crashes, and lower risk of CSI was both found in patients wearing full-coverage or partial-coverage helmets. There were some advantages in our report worth mentioning. Firstly, previous studies reported that the risk of CSI in head injury increased if spinal trauma was present [28], and truncal injury always indicated a serious impact over other anatomic regions [3, 4, 29]. Therefore our finding of the significant association of thoracic and lumbar spine injury with CSI in both Models 1 and 2 supported the construct validity of the present study. Secondly, it was considered that small sample size and heterogeneity in sample composition were the main factors causing controversial results of helmet effect on CSI among previous helmet studies [3, 30]. In order to avoid the lack of statistical power and representation caused by small sample size, we selected 5225 patients from a reliable head injury registry in Taiwan and extracted their data for calculation. We also enrolled only victims of motorcycle crashes with concomitant HI, and all of the motorcycle crashes occurred on the road in the city or countryside, since motorcycles more than 250 cc were not popular and prohibited to be ridden in expressway in Taiwan during the study period. Therefore we believed that bias caused by heterogeneity of sample should be limited.

No evaluation of helmet type was reported in the Crompton study because of the lack of related information in the National Trauma Databank (NTDB) [11]. Full-face helmets had previously been considered to dissipate the force of impact to the trunk and, therefore, reduce the risk of CSI [8]. In Japan, autopsy studies of deceased patients reported that using full-coverage helmets was more likely to result in CSI than using full-face helmets [31]. However, previous studies reported no significant relationship between helmet type and CSI [3, 7, 9, 24, 32]. Our study demonstrated lower risk of CSI following motorcycle crashes in HI patients wearing full-coverage and partial-coverage helmets. We cannot evaluate the effect of full-face helmets because no available information of this helmet type was recorded in the dataset used in this study.

The results of previous biomechanical analyses may explain the phenomenon of lower risk of CSI in HI patients wearing helmet during motorcycle crashes. These biomechanical analyses showed that the excellent flexibility of healthy cervical spine can deflect the head out of the path of the moving torso when the head came into contact with another object. The experiments by using cadaver indicated that CSI caused by head impact was highly correlated with the friction between the head and the surface of the object and surface padding characteristics [33–35]. These 2 factors were strongly influenced by the design and material used in the manufacture of helmets. Low friction on a smooth helmet surface may reduce the peak moment of force exerted on the occiput-C1 joint [33]. The hard linings of padding materials in helmets also reduced the friction between the padding and head and provided additional protection for the cervical spine [34, 35].

There were limitations in this study. Firstly the result of the effect of helmet use on CSI cannot apply to all victims of motorcycle crashes because victims without HI were not included in this study. As retrospective study, the insufficiency of the socioeconomic and environmental information, such as the crashes scenario, including road conditions, vehicle speed, full-face helmet, cord-injury symptoms, was considered as limitation in our study. Since we restricted our study population to northern Taiwan to decrease the bias caused by the aforementioned insufficiency, the result of our study should be used cautiously when applied to the whole country. Although the impact angle, a crucial factor of CSI in head injury, was not included in the dataset [35, 36], the impact angle in the study sample should be homogenous because it exclusively included victims with HI caused by motorcycle crashes. Finally, the reports of National Health Interview Survey conducted by Health Promotion Administration between 2001 and 2009 showed that nearly 90% of motorcyclist used helmet while riding motorcycle [37]; therefore we believed that the bias caused by missing data of helmet use in our original sample should be minimal.

## 5. Conclusion

Using full-coverage and partial-coverage motorcycle helmets exhibited a significantly decreased risk of CSI among HI patients following motorcycle crashes. According to the results of previous biomechanical analyses, the design of helmet, including smooth surface and hard linings of padding materials, was considered as the cause of the effect. Further study should be performed to clarify the effect of full-face helmets on CSI. The result of this study supports helmet use among motorcycle riders.

## Figures and Tables

**Table 1:** Univariate comparison between case and control groups in helmet use, helmet types, and other covariates (*n* = 5225).

Variables	No CSI		CSI		*P* value
	*n* = 5052		*n* = 173		
	*n*	%	*n*	%	
Male	3115	61.8	122	70.5	.021
Age					
17–24	1602	31.7	48	27.7	.232
25–44	1657	32.8	61	35.3	
45–64	1245	24.6	38	22.0	
≧65	548	10.9	26	15.0	
Helmet use^1^	4644	91.9	132	76.3	<.001
Helmet types^2^					
No	408	8.1	41	23.7	<.001
Full coverage	1259	24.9	28	16.2	
Partial coverage	3385	67.0	104	60.1	
Driver	4644	94.4	157	96.3	.282
Ambulance delivery	3920	81.9	148	91.4	.002
Transferred from another hospital	931	19.0	35	20.6	.603
Hospitalization due to HI in the past 5 years	108	2.4	3	1.8	1.000
Glasgow Coma Scale					
13–15	3991	79.5	123	73.2	.130
9–12	504	10.1	21	12.5	
≦8	523	10.4	24	14.3	
HI-induced neurological impairment	386	7.8	27	15.9	<.001
Thoracic spine injury	50	1.0	10	5.8	<.001
Lumbar spine injury	21	0.4	5	2.9	<.001
Skull fracture	655	13.1	26	15.0	.456
Facial fracture	1048	20.7	21	12.1	.006
Chest injury	398	7.9	24	13.9	.004
Abdominal injury	120	2.4	6	3.5	.312

^1^Only for Model 1.

^2^Only for Model 2.

CSI: cervical spine injury; HI: head injury.

**Table 2:** Multiple logistic regression analyses between case and control groups in helmet use and other covariates and the odds ratio of CSI (Model 1).

Variables	OR	95% CI for OR		*P* value
		Lower	Upper	
Helmet use				
No	1.00	—	—	
Yes	0.31	0.19	0.49	<.001
Method of transport to the hospital				
Self-transportation	1.00	—	—	
Ambulance delivery	1.99	1.08	3.67	.028
Thoracic spine injury				
No	1.00	—	—	
Yes	4.70	2.00	11.05	<.001
Lumbar spine injury				
No	1.00	—	—	
Yes	4.82	1.47	15.86	.010

OR: odds ratio; CI: confidence interval; CSI: cervical spine injury; and HI: head injury.

The dependent variable in Model 1 was CSI and the independent variables included all variables in Table 1 except helmet type. Only the significant variables (*P* < .05) were shown in Table 2.

**Table 3:** Multiple logistic regression analyses between case and control groups in helmet types and other covariates and the odds ratio of CSI (Model 2).

Variables	OR	95% CI for OR		*P* value
		Lower	Upper	
Helmet types				
No	1.00	—	—	
Full coverage	0.19	0.10	0.36	<.001
Partial coverage	0.35	0.21	0.56	<.001
Thoracic spine injury				
No	1.00	—	—	
Yes	4.46	1.89	10.48	<.001
Lumbar spine injury				
No	1.00	—	—	
Yes	4.87	1.46	16.23	.010

OR: odds ratio; CI: confidence interval; CSI: cervical spine injury; and HI: head injury.

The dependent variable in Model 2 was CSI and the independent variables included all variables in Table 1 except helmet use. Only the significant variables (*P* < .05) were shown in Table 3.
